# (2-Meth­oxy-1,10-phenanthroline-κ^2^ *N*,*N*′)bis­(thio­cyanato-κ*N*)zinc(II)

**DOI:** 10.1107/S1600536808011793

**Published:** 2008-05-03

**Authors:** Hong Li, Tai Qiu Hu, Shi Guo Zhang

**Affiliations:** a Department of Chemistry and Chemical Engineering, Institute of Materials Chemistry, Binzhou University, Binzhou 256603, People’s Republic of China; b Department of Chemistry, Shandong Normal University, Jinan 250014, People’s Republic of China

## Abstract

In the title complex, [Zn(NCS)_2_(C_13_H_10_N_2_O)], the Zn^II^ ion is in a distorted tetra­hdral ZnN_2_Cl_2_ coordination environment. In the crystal structure, there is a weak π–π stacking inter­action between adjacent 1,10-phenanthroline rings, with a pyridine centroid–centroid distance of 3.6620 (15) Å.

## Related literature

For a related structure, see: Zhang *et al.* (2006 ▶). For related literature, see: McMorran & Steel (2002 ▶).
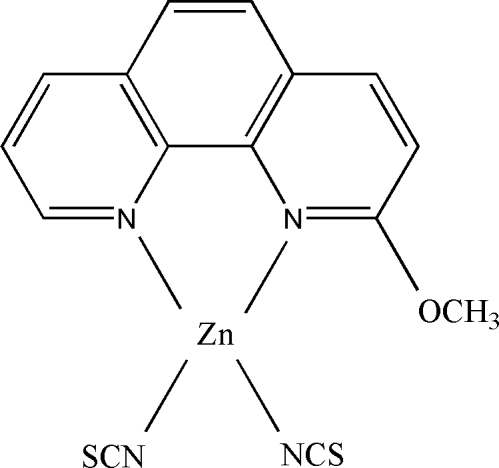


## Experimental

### 

#### Crystal data

[Zn(NCS)_2_(C_13_H_10_N_2_O)]
*M* *_r_* = 391.76
Monoclinic, 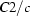

*a* = 26.360 (5) Å
*b* = 8.5949 (16) Å
*c* = 14.814 (3) Å
β = 96.266 (2)°
*V* = 3336.3 (10) Å^3^
*Z* = 8
Mo *K*α radiation
μ = 1.73 mm^−1^
*T* = 298 (2) K
0.61 × 0.42 × 0.40 mm

#### Data collection

Bruker SMART APEX CCD diffractometer
Absorption correction: multi-scan (*SADABS*; Sheldrick, 1996 ▶) *T*_min_ = 0.418, *T*_max_ = 0.545 (expected range = 0.385–0.501)
9311 measured reflections
3616 independent reflections
2974 reflections with *I* > 2σ(*I*)
*R*_int_ = 0.040

#### Refinement

*R*[*F* ^2^ > 2σ(*F* ^2^)] = 0.035
*wR*(*F* ^2^) = 0.097
*S* = 1.05
3616 reflections
209 parameters
H-atom parameters constrained
Δρ_max_ = 0.33 e Å^−3^
Δρ_min_ = −0.49 e Å^−3^

### 

Data collection: *SMART* (Bruker, 1997 ▶); cell refinement: *SAINT* (Bruker, 1997 ▶); data reduction: *SAINT*; program(s) used to solve structure: *SHELXTL* (Sheldrick, 2008 ▶); program(s) used to refine structure: *SHELXTL*; molecular graphics: *SHELXTL*; software used to prepare material for publication: *SHELXTL*.

## Supplementary Material

Crystal structure: contains datablocks I, global. DOI: 10.1107/S1600536808011793/lh2603sup1.cif

Structure factors: contains datablocks I. DOI: 10.1107/S1600536808011793/lh2603Isup2.hkl

Additional supplementary materials:  crystallographic information; 3D view; checkCIF report

## Figures and Tables

**Table:** 

Zn1—N3	1.916 (2)
Zn1—N4	1.926 (2)
Zn1—N2	2.0254 (16)
Zn1—N1	2.0636 (19)

**Table:** 

N3—Zn1—N4	114.85 (9)
N3—Zn1—N2	116.36 (8)
N4—Zn1—N2	115.23 (9)
N3—Zn1—N1	116.20 (8)
N4—Zn1—N1	108.07 (8)
N2—Zn1—N1	81.62 (7)

**Fig. 1.**
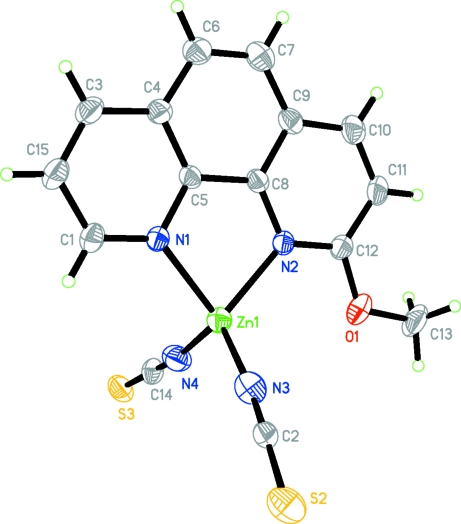
The molecular structure of (I), showing the the atom numbering scheme with thermal ellipsoids drawn at the 30% probability level.

## supplementary crystallographic information

### Comment

Derivatives of 1,10-phenanthroline play a pivotal role in the area of modern coordination chemistry (e.g. Zhang *et al.* 2006 and important references cited within), but no structures of complexes with 2-methoxy-1,10-phenanthroline as a ligand have been reported. Herein we report the crystal structure of the title complex (I).

The molecular structure of (I) is shown in Fig. 1. In this mononuclear complex atom Zn1 is in a distorted tetrahedral coordination geometry (Table 1). In the crystal structure, there are weak π-π stacking interactions between symmetry related 1,10-phenanthroline ligands, with the relevant distances being *Cg*1···*Cg*1^i^ = 3.6620 (15) Å and a perpendicular distance of 3.563 Å [symmetry code: (i) 1/2-*x*, 3/2-y, 1-*z*; *Cg*1 is the centroid of the N1/C1/C3/C4/C5/C15 ring].

### Experimental

A methanol solution (15ml) of Zn(ClO_4_).6H_2_O (0.2951 g, 0.792 mmol) was added into a 10 ml methanol solution containing 2-methoxy-1,10-phenanthroline (0.1666 g, 0.792 mmol), and the mixture was stirred for a few minutes. Then a 10 ml methanol solution of NaSCN (0.1296 g, 1.60 mmol) was added to the above mixture. Yellow single crystals were obtained after the solution had been allowed to stand at room temperature for two weeks.

### Refinement

H atoms were placed in calculated positions (C—H = 0.96 Å for methyl group and C—H = 0.93 Å for other H atoms) and refined as riding with *U*_iso_ = 1.5 *U*_eq_(C) for methyl H and *U*_iso_ = 1.2 *U*_eq_(C) for other H.

### Crystal data

**Table:**

[Zn(NCS)_2_(C_13_H_10_N_2_O)]	*F*_000_ = 1584
*M**_r_* = 391.76	*D*_x_ = 1.560 Mg m^−^^3^
Monoclinic, *C*2/*c*	Mo *K*α radiation λ = 0.71073 Å
Hall symbol: -C 2yc	Cell parameters from 4025 reflections
*a* = 26.360 (5) Å	θ = 2.2–27.6º
*b* = 8.5949 (16) Å	µ = 1.73 mm^−^^1^
*c* = 14.814 (3) Å	*T* = 298 (2) K
β = 96.266 (2)º	Bar, yellow
*V* = 3336.3 (10) Å^3^	0.61 × 0.42 × 0.40 mm
*Z* = 8	

### Data collection

**Table:**

Bruker SMART APEX CCD diffractometer	3616 independent reflections
Radiation source: fine-focus sealed tube	2974 reflections with *I* > 2σ(*I*)
Monochromator: graphite	*R*_int_ = 0.040
*T* = 298(2) K	θ_max_ = 27.0º
φ and ω scans	θ_min_ = 2.5º
Absorption correction: multi-scan(SADABS; Sheldrick, 1996)	*h* = −33→26
*T*_min_ = 0.418, *T*_max_ = 0.545	*k* = −9→10
9311 measured reflections	*l* = −18→18

### Refinement

**Table:**

Refinement on *F*^2^	Secondary atom site location: difference Fourier map
Least-squares matrix: full	Hydrogen site location: inferred from neighbouring sites
*R*[*F*^2^ > 2σ(*F*^2^)] = 0.035	H-atom parameters constrained
*wR*(*F*^2^) = 0.097	*w* = 1/[σ^2^(*F*_o_^2^) + (0.0536*P*)^2^ + 0.2305*P*] where *P* = (*F*_o_^2^ + 2*F*_c_^2^)/3
*S* = 1.06	(Δ/σ)_max_ = 0.001
3616 reflections	Δρ_max_ = 0.33 e Å^−^^3^
209 parameters	Δρ_min_ = −0.49 e Å^−^^3^
Primary atom site location: structure-invariant direct methods	Extinction correction: none

### Special details

**Table:**

Geometry. All e.s.d.'s (except the e.s.d. in the dihedral angle between two l.s. planes) are estimated using the full covariance matrix. The cell e.s.d.'s are taken into account individually in the estimation of e.s.d.'s in distances, angles and torsion angles; correlations between e.s.d.'s in cell parameters are only used when they are defined by crystal symmetry. An approximate (isotropic) treatment of cell e.s.d.'s is used for estimating e.s.d.'s involving l.s. planes.
Refinement. Refinement of *F*^2^ against ALL reflections. The weighted *R*-factor *wR* and goodness of fit *S* are based on *F*^2^, conventional *R*-factors *R* are based on *F*, with *F* set to zero for negative *F*^2^. The threshold expression of *F*^2^ > σ(*F*^2^) is used only for calculating *R*-factors(gt) *etc*. and is not relevant to the choice of reflections for refinement. *R*-factors based on *F*^2^ are statistically about twice as large as those based on *F*, and *R*- factors based on ALL data will be even larger.

### Fractional atomic coordinates and isotropic or equivalent isotropic displacement parameters (Å^2^)

**Table:**

	*x*	*y*	*z*	*U*_iso_*/*U*_eq_	
Zn1	0.130923 (10)	1.01266 (3)	0.392499 (16)	0.04081 (11)	
S2	0.07232 (4)	1.52220 (8)	0.35198 (6)	0.0700 (2)	
S3	0.15576 (3)	0.73246 (10)	0.13159 (4)	0.0627 (2)	
N1	0.19776 (7)	0.98293 (19)	0.47711 (12)	0.0374 (4)	
N2	0.10343 (6)	0.8750 (2)	0.48692 (10)	0.0372 (4)	
C5	0.19054 (7)	0.8893 (2)	0.54870 (12)	0.0350 (4)	
N3	0.10454 (8)	1.2204 (3)	0.38239 (13)	0.0557 (5)	
O1	0.02497 (6)	0.8745 (2)	0.41868 (12)	0.0593 (4)	
C4	0.22974 (8)	0.8481 (3)	0.61569 (13)	0.0415 (5)	
C8	0.13985 (8)	0.8311 (2)	0.55315 (13)	0.0363 (4)	
C6	0.21868 (10)	0.7477 (3)	0.68714 (15)	0.0510 (6)	
H6	0.2446	0.7205	0.7320	0.061*	
C12	0.05651 (8)	0.8226 (3)	0.48815 (15)	0.0446 (5)	
N4	0.13885 (8)	0.9127 (3)	0.27851 (13)	0.0580 (5)	
C14	0.14584 (8)	0.8383 (3)	0.21771 (14)	0.0424 (5)	
C9	0.13065 (9)	0.7316 (3)	0.62413 (15)	0.0435 (5)	
C2	0.09073 (8)	1.3458 (3)	0.37012 (13)	0.0422 (5)	
C7	0.17119 (10)	0.6909 (3)	0.69101 (15)	0.0530 (6)	
H7	0.1650	0.6244	0.7381	0.064*	
C11	0.04328 (10)	0.7227 (3)	0.55773 (17)	0.0552 (6)	
H11	0.0099	0.6878	0.5580	0.066*	
C3	0.27856 (9)	0.9092 (3)	0.60652 (16)	0.0517 (6)	
H3	0.3059	0.8854	0.6494	0.062*	
C10	0.07972 (10)	0.6789 (3)	0.62360 (16)	0.0545 (6)	
H10	0.0713	0.6130	0.6695	0.065*	
C15	0.28561 (10)	1.0030 (3)	0.53490 (19)	0.0543 (7)	
H15	0.3177	1.0435	0.5286	0.065*	
C1	0.24425 (9)	1.0379 (3)	0.47095 (17)	0.0471 (5)	
H1	0.2495	1.1020	0.4223	0.057*	
C13	−0.02649 (9)	0.8142 (4)	0.4045 (2)	0.0721 (8)	
H13A	−0.0254	0.7027	0.4012	0.108*	
H13B	−0.0433	0.8548	0.3487	0.108*	
H13C	−0.0449	0.8450	0.4540	0.108*	

### Atomic displacement parameters (Å^2^)

**Table:**

	*U*^11^	*U*^22^	*U*^33^	*U*^12^	*U*^13^	*U*^23^
Zn1	0.03960 (18)	0.04250 (18)	0.04019 (16)	0.00072 (11)	0.00369 (11)	0.00223 (10)
S2	0.0862 (6)	0.0478 (4)	0.0775 (5)	0.0193 (4)	0.0155 (4)	−0.0028 (3)
S3	0.0606 (4)	0.0723 (5)	0.0568 (4)	0.0133 (3)	0.0135 (3)	−0.0126 (3)
N1	0.0323 (10)	0.0394 (10)	0.0409 (9)	−0.0019 (7)	0.0053 (7)	−0.0012 (7)
N2	0.0320 (9)	0.0377 (10)	0.0427 (9)	−0.0020 (8)	0.0078 (7)	−0.0029 (7)
C5	0.0357 (11)	0.0319 (10)	0.0383 (10)	0.0010 (9)	0.0080 (8)	−0.0052 (8)
N3	0.0631 (14)	0.0480 (13)	0.0566 (12)	0.0108 (11)	0.0095 (10)	0.0064 (9)
O1	0.0332 (9)	0.0696 (12)	0.0735 (11)	−0.0053 (8)	−0.0016 (7)	0.0038 (9)
C4	0.0399 (12)	0.0424 (12)	0.0419 (11)	0.0026 (10)	0.0027 (9)	−0.0043 (9)
C8	0.0378 (11)	0.0344 (11)	0.0380 (10)	0.0001 (9)	0.0099 (8)	−0.0028 (8)
C6	0.0518 (15)	0.0577 (15)	0.0419 (12)	0.0095 (12)	−0.0021 (10)	0.0045 (10)
C12	0.0355 (12)	0.0456 (13)	0.0533 (12)	−0.0043 (10)	0.0072 (9)	−0.0080 (10)
N4	0.0637 (14)	0.0639 (14)	0.0467 (11)	0.0039 (12)	0.0065 (9)	−0.0073 (11)
C14	0.0345 (11)	0.0474 (13)	0.0448 (12)	0.0044 (10)	0.0024 (9)	0.0073 (10)
C9	0.0524 (14)	0.0397 (12)	0.0406 (11)	−0.0023 (10)	0.0145 (9)	0.0000 (9)
C2	0.0395 (12)	0.0512 (14)	0.0369 (10)	0.0001 (11)	0.0089 (8)	−0.0035 (9)
C7	0.0645 (17)	0.0512 (15)	0.0451 (12)	0.0054 (12)	0.0135 (11)	0.0098 (11)
C11	0.0421 (13)	0.0618 (16)	0.0648 (15)	−0.0143 (12)	0.0205 (11)	−0.0046 (12)
C3	0.0390 (12)	0.0560 (15)	0.0572 (13)	0.0013 (11)	−0.0072 (10)	−0.0045 (12)
C10	0.0574 (15)	0.0576 (15)	0.0522 (13)	−0.0120 (13)	0.0220 (11)	0.0036 (11)
C15	0.0366 (13)	0.0585 (17)	0.0672 (17)	−0.0106 (11)	0.0034 (12)	−0.0008 (12)
C1	0.0373 (13)	0.0486 (13)	0.0561 (13)	−0.0088 (11)	0.0083 (10)	0.0019 (10)
C13	0.0303 (13)	0.086 (2)	0.099 (2)	−0.0058 (14)	−0.0020 (13)	−0.0092 (17)

### Geometric parameters (Å, °)

**Table:**

Zn1—N3	1.916 (2)	C6—C7	1.351 (3)
Zn1—N4	1.926 (2)	C6—H6	0.9300
Zn1—N2	2.0254 (16)	C12—C11	1.414 (3)
Zn1—N1	2.0636 (19)	N4—C14	1.136 (3)
S2—C2	1.606 (3)	C9—C10	1.416 (3)
S3—C14	1.611 (2)	C9—C7	1.419 (3)
N1—C1	1.326 (3)	C7—H7	0.9300
N1—C5	1.361 (3)	C11—C10	1.346 (4)
N2—C12	1.319 (3)	C11—H11	0.9300
N2—C8	1.349 (3)	C3—C15	1.361 (4)
C5—C4	1.398 (3)	C3—H3	0.9300
C5—C8	1.435 (3)	C10—H10	0.9300
N3—C2	1.145 (3)	C15—C1	1.397 (4)
O1—C12	1.328 (3)	C15—H15	0.9300
O1—C13	1.446 (3)	C1—H1	0.9300
C4—C3	1.410 (3)	C13—H13A	0.9600
C4—C6	1.421 (3)	C13—H13B	0.9600
C8—C9	1.397 (3)	C13—H13C	0.9600
			
N3—Zn1—N4	114.85 (9)	N4—C14—S3	179.9 (3)
N3—Zn1—N2	116.36 (8)	C8—C9—C10	115.7 (2)
N4—Zn1—N2	115.23 (9)	C8—C9—C7	119.8 (2)
N3—Zn1—N1	116.20 (8)	C10—C9—C7	124.5 (2)
N4—Zn1—N1	108.07 (8)	N3—C2—S2	178.9 (2)
N2—Zn1—N1	81.62 (7)	C6—C7—C9	120.8 (2)
C1—N1—C5	118.2 (2)	C6—C7—H7	119.6
C1—N1—Zn1	130.42 (16)	C9—C7—H7	119.6
C5—N1—Zn1	111.33 (14)	C10—C11—C12	119.0 (2)
C12—N2—C8	119.23 (18)	C10—C11—H11	120.5
C12—N2—Zn1	128.04 (15)	C12—C11—H11	120.5
C8—N2—Zn1	112.68 (13)	C15—C3—C4	119.9 (2)
N1—C5—C4	123.20 (19)	C15—C3—H3	120.0
N1—C5—C8	116.87 (18)	C4—C3—H3	120.0
C4—C5—C8	119.93 (18)	C11—C10—C9	121.0 (2)
C2—N3—Zn1	174.4 (2)	C11—C10—H10	119.5
C12—O1—C13	119.4 (2)	C9—C10—H10	119.5
C5—C4—C3	116.7 (2)	C3—C15—C1	119.5 (2)
C5—C4—C6	119.1 (2)	C3—C15—H15	120.2
C3—C4—C6	124.2 (2)	C1—C15—H15	120.2
N2—C8—C9	123.38 (19)	N1—C1—C15	122.4 (2)
N2—C8—C5	117.48 (17)	N1—C1—H1	118.8
C9—C8—C5	119.14 (19)	C15—C1—H1	118.8
C7—C6—C4	121.2 (2)	O1—C13—H13A	109.5
C7—C6—H6	119.4	O1—C13—H13B	109.5
C4—C6—H6	119.4	H13A—C13—H13B	109.5
N2—C12—O1	112.55 (19)	O1—C13—H13C	109.5
N2—C12—C11	121.6 (2)	H13A—C13—H13C	109.5
O1—C12—C11	125.8 (2)	H13B—C13—H13C	109.5
C14—N4—Zn1	171.4 (2)		
			
N3—Zn1—N1—C1	−64.6 (2)	C4—C5—C8—C9	−1.0 (3)
N4—Zn1—N1—C1	66.2 (2)	C5—C4—C6—C7	0.4 (3)
N2—Zn1—N1—C1	−179.9 (2)	C3—C4—C6—C7	−178.8 (2)
N3—Zn1—N1—C5	116.32 (14)	C8—N2—C12—O1	−179.57 (18)
N4—Zn1—N1—C5	−112.90 (14)	Zn1—N2—C12—O1	−2.3 (3)
N2—Zn1—N1—C5	1.00 (13)	C8—N2—C12—C11	0.7 (3)
N3—Zn1—N2—C12	66.0 (2)	Zn1—N2—C12—C11	177.98 (16)
N4—Zn1—N2—C12	−72.75 (19)	C13—O1—C12—N2	173.2 (2)
N1—Zn1—N2—C12	−178.84 (19)	C13—O1—C12—C11	−7.1 (4)
N3—Zn1—N2—C8	−116.55 (14)	N2—C8—C9—C10	−0.6 (3)
N4—Zn1—N2—C8	104.70 (15)	C5—C8—C9—C10	−179.83 (19)
N1—Zn1—N2—C8	−1.40 (13)	N2—C8—C9—C7	179.9 (2)
C1—N1—C5—C4	−0.2 (3)	C5—C8—C9—C7	0.6 (3)
Zn1—N1—C5—C4	179.00 (16)	C4—C6—C7—C9	−0.7 (4)
C1—N1—C5—C8	−179.68 (19)	C8—C9—C7—C6	0.2 (4)
Zn1—N1—C5—C8	−0.5 (2)	C10—C9—C7—C6	−179.3 (2)
N1—C5—C4—C3	0.2 (3)	N2—C12—C11—C10	−0.8 (4)
C8—C5—C4—C3	179.70 (19)	O1—C12—C11—C10	179.5 (2)
N1—C5—C4—C6	−178.99 (19)	C5—C4—C3—C15	−0.1 (3)
C8—C5—C4—C6	0.5 (3)	C6—C4—C3—C15	179.1 (2)
C12—N2—C8—C9	0.1 (3)	C12—C11—C10—C9	0.2 (4)
Zn1—N2—C8—C9	−177.64 (16)	C8—C9—C10—C11	0.5 (3)
C12—N2—C8—C5	179.27 (18)	C7—C9—C10—C11	180.0 (2)
Zn1—N2—C8—C5	1.6 (2)	C4—C3—C15—C1	0.0 (4)
N1—C5—C8—N2	−0.7 (3)	C5—N1—C1—C15	0.1 (3)
C4—C5—C8—N2	179.77 (18)	Zn1—N1—C1—C15	−178.98 (17)
N1—C5—C8—C9	178.51 (17)	C3—C15—C1—N1	0.1 (4)
